# Genomic Analysis of Historical Cases with Positive Newborn Screens for Short-Chain Acyl-CoA Dehydrogenase Deficiency Shows That a Validated Second-Tier Biochemical Test Can Replace Future Sequencing

**DOI:** 10.3390/ijns6020041

**Published:** 2020-05-26

**Authors:** Aashish N. Adhikari, Robert J. Currier, Hao Tang, Coleman T. Turgeon, Robert L. Nussbaum, Rajgopal Srinivasan, Uma Sunderam, Pui-Yan Kwok, Steven E. Brenner, Dimitar Gavrilov, Jennifer M. Puck, Renata Gallagher

**Affiliations:** 1 Department of Plant and Microbial Biology, University of California, Berkeley, CA 94720, USA; anadhikari@berkeley.edu (A.N.A.); brenner@compbio.berkeley.edu (S.E.B.); 2 Department of Pediatrics, University of California, San Francisco, CA 94158, USA; jennifer.puck@ucsf.edu; 3 Genetic Disease Screening Program, California Department of Public Health, Richmond, CA 94804, USA; hao.tang@cdph.ca.gov; 4 Biochemical Genetics Laboratory, Department of Laboratory Medicine and Pathology, Mayo Clinic, Rochester, MN 55905, USA; turgeon.coleman@mayo.edu (C.T.T.); gavrilov.dimitar@mayo.edu (D.G.); 5 Invitae, San Francisco, CA 94103, USA; robert.nussbaum@invitae.com; 6 Innovation Labs, Tata Consultancy Services, Hyderabad 500081, India; rajgopal.srinivasan@tcs.com (R.S.); uma.sunderam@tcs.com (U.S.); 7 Institute for Human Genetics, University of California, San Francisco, CA 94153, USA; pui.kwok@ucsf.edu; 8 Cardiovascular Research Institute, University of California, San Francisco, CA 94153, USA; 9 Department of Dermatology, University of California, San Francisco, CA 94153, USA; 10 Center for Computational Biology, University of California, Berkeley, CA 94720, USA; 11 Department of Bioengineering and Therapeutic Sciences, University of California, San Francisco, CA 94158, USA; 12 Department of Clinical Genomics, Mayo Clinic, Rochester, MN 55905, USA; 13 UCSF Benioff Children’s Hospital, Division of Allergy, Immunology and Blood and Marrow Transplantation, San Francisco, CA 94153, USA

**Keywords:** newborn screening, short-chain acyl-CoA dehydrogenase deficiency, SCADD, *ACADS*, second-tier testing, ethylmalonic acid, exome sequence, butyrylcarnitine

## Abstract

Short-chain acyl-CoA dehydrogenase deficiency (SCADD) is a rare autosomal recessive disorder of β-oxidation caused by pathogenic variants in the *ACADS* gene. Analyte testing for SCADD in blood and urine, including newborn screening (NBS) using tandem mass spectrometry (MS/MS) on dried blood spots (DBSs), is complicated by the presence of two relatively common *ACADS* variants (c.625G>A and c.511C>T). Individuals homozygous for these variants or compound heterozygous do not have clinical disease but do have reduced short-chain acyl-CoA dehydrogenase (SCAD) activity, resulting in elevated blood and urine metabolites. As part of a larger study of the potential role of exome sequencing in NBS in California, we reviewed *ACADS* sequence and MS/MS data from DBSs from a cohort of 74 patients identified to have SCADD. Of this cohort, approximately 60% had one or more of the common variants and did not have the two rare variants, and thus would need no further testing. Retrospective analysis of ethylmalonic acid, glutaric acid, 2-hydroxyglutaric acid, 3-hydroxyglutaric acid, and methylsuccinic acid demonstrated that second-tier testing applied before the release of the newborn screening result could reduce referrals by over 50% and improve the positive predictive value for SCADD to above 75%.

## 1. Introduction

Short-chain acyl-CoA dehydrogenase deficiency (SCADD, OMIM:201470) is a rare autosomal recessive disorder of β-oxidation caused by pathogenic variants in the *ACADS* gene. Historically, the reported spectrum of disease included an infantile presentation with metabolic acidosis, failure to thrive, and seizures; and a late-onset form with chronic myopathy. The biochemical signatures of short-chain acyl-CoA dehydrogenase (SCAD) include elevated plasma butyrylcarnitine (C4) and urine ethylmalonic acid (EMA) [1].

Analyte testing for SCAD disease (SCADD) in blood and urine is complicated by the high allele frequency of two *ACADS* variants (c.625G>A, p.Gly209Ser (rs1799958) and c.511C>T, p.Arg171Trp (rs1800556) (NM_000017.3)). Individuals who are homozygotes for either of these variants or who are compound heterozygotes do not have clinical disease, but have reduced SCAD enzyme activity, resulting in elevated blood and urine metabolites. These variants have allele frequencies of 0.2577 (ranging from 0.1252 in East Asians to 0.3660 in Latino populations) and 0.03110 (0.000 in East Asians to 0.06326 in the Finnish population), respectively, [2] (population frequencies from gnomAD, variants 12-121176083-G-A and 12-121175678-C-T, respectively).

In an early study, newborns who were homozygous or compound heterozygous for these variants had dried blood spot (DBS) C4 acylcarnitine levels below the cutoff of 1 µmol/L [3], and therefore, individuals with these variants were not expected to be identified through newborn screening (NBS) by tandem mass spectrometry (MS/MS), which assesses this analyte as a marker for SCADD, and other disorders.

In 2006, the American College of Medical Genetics published recommendations for a uniform panel for NBS in the United States [4]. Among criteria for inclusion in the panel were the availability of a screening test with appropriate sensitivity and specificity, severe disease phenotype, and demonstrated benefits of early detection, timely intervention, and efficacious treatment. Based on numerical scores applied to these criteria, and what was then understood about the condition, SCADD ranked in the middle of the 84 conditions evaluated, scoring slightly above cystic fibrosis. SCADD was included as a secondary condition on the list of the Recommended Uniform Screening Panel (RUSP) of the Advisory Committee on Heritable Disorders of Newborns and Children (ACHDNC) as a condition that is identified incidentally in the interpretation of the MS/MS profile. SCADD is one of three disorders identified by MS/MS from elevated butyrylcarnitine (C4 acylcarnitine) [5].

Through NBS and clinical follow-up of children with abnormal screening results, the natural history of SCADD has been further elucidated in the years since 2006. In many cases, these individuals have been asymptomatic, raising the question of whether SCADD is a true disease [6,7]. As a result, some NBS programs have removed SCADD from their screening panels [8], though C4 acylcarnitine also aids in the identification of other disorders, including, multiple acyl-CoA dehydrogenase deficiency (MADD) and isobutyryl-CoA dehydrogenase deficiency (IBDD). Some programs have been reluctant to stop evaluating newborns with elevated C4 acylcarnitine in order not to miss these. Complicating the evaluation of newborns with elevated C4 acylcarnitine are the false positive results due to the *ACADS* gene variants described above, which do not cause overt disease. Some of the compound heterozygotes or homozygotes for these variants have abnormal newborn screening results by MS/MS, in some cases requiring extensive and costly follow-up testing to exclude disease. This situation creates a burden on the follow-up metabolic centers, unnecessary anxiety for families, and unnecessary intervention (fasting precautions and emergency management of any symptoms of infection) for unaffected newborns. In many cases, DNA sequencing is required to confirm or rule out true SCADD, and to exclude other disorders in the differential diagnosis. True SCADD is confirmed by demonstration of two known or likely pathogenic variants in *trans* in the *ACADS* gene, in addition to high urinary ethylmalonic acid and high plasma C4 acylcarntine [1].

The goal of this study was to assess the correlation of the initial DBS C4-acylcarnitine value, the second-tier DBS ethylmalonic acid value, and exome sequencing results in a large cohort of individuals identified as potential cases of SCADD through the California newborn screening program. We hypothesized that combined consideration of multiple test results could improve the specificity of newborn screening for SCADD and other disorders identified though C4 acylcarnitine levels, thereby potentially reducing the burden of follow-up testing and management.

Powerful tools for the interpretation of MS/MS profiles, with or without the inclusion of second-tier testing results are provided by the Collaborative Laboratory Integrated Reports (CLIR) developed at the Mayo Clinic [9]. In CLIR, the single condition tool for SCAD interpretation considers not only C4, but also fifteen additional ratios to provide better specificity in interpretation [10].

The current study was conducted as part of the NBSeq project, which assessed the role of DNA sequencing in NBS, taking advantage of deidentified, stored DBSs from the California NBS program from which both additional DNA and analyte testing could be obtained with appropriate scientific and research protection review and approval [11]. DBSs were obtained from individuals diagnosed with SCADD in California through newborn screening and follow-up laboratory testing between mid-2005 and 2013.

### Terminology in CLIR

Current practice recommends referring to each nucleotide difference from the reference sequence of a gene as a variant. In prior nomenclature, variants that were present in >4% of the population were called “polymorphisms” and were understood to be benign. The SCAD common variants described above are sometimes called susceptibility alleles [1]. Here, we refer to the *ACADS* susceptibility alleles as polymorphisms denoted as P, and rare variants identified in the NBSeq pipeline as mutations denoted as M. Genotypes identified in this work are recorded for each case as a combination of one or more polymorphisms and one or more mutations (for novel missense variants the true pathogenicity is unknown). For example, SCAD(2P) indicates apparent homozygous c.625G>A, c.511C>T, while the presence of SCAD(1M1P) indicates the presence of one of the common variants with a rare variant, and SCAD(2M) indicates the presence of two rare variants with the potential to confer disease. Note that is it not possible to determine whether the identified polymorphisms and mutations are present on the same or the opposite chromosomes (i.e., the phase of the variants cannot be identified by exome sequencing, unless additional studies are performed, such as parental sequencing).

## 2. Materials and Methods

This work was conducted through the NBSeq study, described separately [12]. Briefly, de-identified, archived DBS specimens stored desiccated at −20 °C were obtained from the California Biobank Program consisting of all confirmed cases of inborn errors of metabolism (IEM) diagnosed by tandem mass spectrometry between mid-2005 and 2013. DNA was prepared from the DBSs and sequenced as described, and an “exome slice” of genes relevant to the screened disorders was analyzed. An interpretive pipeline identified known pathogenic variants, presumed protein-altering variants (stop gain, stop loss, frame-shifting indels, splice site alteration), and rare missense variants. Predicted disease status was based on the identification of one or two “reportable” variants, depending on the mode of inheritance of each disease. Predictions from the pipeline were compared with clinical status recorded in the follow-up database of the California Genetic Disease Screening Program (GDSP).

The DNA analysis pipeline developed for screening purposes, which did not include the *ACADS* polymorphisms as reportable variants, failed to identify multiple cases clinically diagnosed through newborn screening by tandem mass spectrometry and follow-up testing as SCADD. This was presumed to be due to the newborn screening centers’ practice of reporting individuals with one or more polymorphisms as true SCADD, either as they lacked the sequence information, or used a more conservative definition of SCADD that included individuals with one or two polymorphisms. We sought in this study to assess whether additional biochemical information could aid in case resolution without follow-up testing by the metabolic center.

### Second-Tier Biochemical Assay: Hydroxyglutaric ACIDS, Glutaric Acid, Ethylmalonic Acid, and Methylsuccinic Acid (HGEM)

To a 3 mm DBS, methanol containing isotopically labeled internal standards was added. The mixture was rotated at ambient conditions for 2 h, and the eluate was transferred to a second 96-well plate. Methanol was removed under heated nitrogen with the resulting residue then reconstituted with water. Prepared samples were analyzed by reverse-phase liquid chromatography-tandem mass spectrometry (LC-MS/MS). The MS/MS was operated in the multiple reaction monitoring (MRM) negative mode to follow the precursor to product species transitions for 2-hydroxyglutaric acid (2OH-GA) (147 to 129 *m*/*z*), 3-hydroxyglutaric acid (3OH-GA) (147 to 85 *m*/*z*), glutaric acid (GA), ethylmalonic acid (EMA), and methylsuccinic acid (MSA) (131 to 87 *m*/*z*) as well as their corresponding isotopically labeled internal standards. Total run time was 23 min per sample.

In order to perform the second-tier analyte testing, additional 3 mm punches from DBSs from the SCADD-affected cohort were requested from the California Biobank Program and were sent to the Biochemical Genetics Laboratory of the Mayo Clinic for the HGEM analysis [13]. This is the standard second-tier test at the Biochemical Genetics Laboratory of the Mayo Clinic for newborn screening with elevated C4, but it had not been used in California at the time of the original newborn screening. In addition, the excel file of the initial MS/MS data from newborn screening was sent to the Mayo Clinic for evaluation using the CLIR tools for SCADD. Subsequent to the second-tier analysis of the DBSs, CLIR tools for SCADD were created including the second-tier analytes. Initially, the *ACADS* genotype was not included in the data sent to the Mayo Clinic during the evaluation of these cases using the CLIR tools. Subsequently, the second-tier CLIR tool was further refined after unblinding the genotype results of the SCADD cohort.

## 3. Results

The initial pipeline evaluation of the *ACADS* genotypes found that, of the 74 California newborn screen cases reported to have SCADD identified in the study period, 30 had two rare variants (2M or 2M1P or 2M2P), 31 had only one rare variant (1M1P or 1M2P), and 13 had no rare variants (2P or 1P or 0P) (Table 1, variants in cases are listed in Table S1). Based on the current understanding that only cases with two rare variants should be considered SCADD, the positive predictive value (PPV) of the original MS/MS screen for SCADD among the cases considered SCADD at the time of testing would be 40.5%.

CLIR tools used to evaluate the initial MS/MS screening results found that 25 of the 74 cases clinically diagnosed as SCADD (33.8%) were unlikely to be SCAD(2M). Only five clinical cases were identified by CLIR as likely true SCAD(2M) based on the initial MS/MS result alone, while the remaining 44 were characterized as indeterminate. For the latter, the CLIR tools suggested performing the second-tier test (2TT) (Table 2).

The Mayo Clinic second-tier test for ethylmalonic acid, glutaric acid, 2-hydroxyglutaric acid, 3-hydroxyglutaric acid, and methylsuccinic acid was run on the additional punched specimens from the initial DBSs for all the California SCADD retrospective clinical cases. CLIR tools were developed that incorporated these analytes in addition to the initial MS/MS analytes. These tools identified 43 cases as likely SCAD(2M), 26 as likely not SCAD(2M), and only 5 as remaining uncertain (Table 3). In NBS performed by the Mayo Biochemical Genetics Laboratory using second-tier testing and CLIR, the uncertain category would be reported out as requiring clinical follow-up, so 35.1% of the initial positive results would have been eliminated by the second-tier test, with the PPV increased to 62.5%. At this stage, the genotype information had not been revealed.

The CLIR assessment utilizing the second-tier test results was then evaluated with the *ACADS* genotype. Ethylmalonic acid is the diagnostic metabolite in SCADD, and it can be quite high in SCAD(1MxP). The distributions of C4 and EMA by genotype category are show in Figure S1, panels A and B. Panels C and D show the scatter plots of C4 versus EMA, with panel D focusing on the SCAD(1M1P) and showing the rare variant in those cases with elevated EMA. Raising the cutoff for EMA allowed better separation between SCAD(2M) and the not SCAD(2M) cases (Table 4). The revised tools identified 30 cases as SCAD(2M), 36 cases as not SCAD(2M), and the remaining 8 remained in the uncertain category, a reduction of 48.6% of the initial positives that had been clinically identified as SCADD, and an increase of the PPV to 78.9%. An additional feature of this analysis was the observation that SCAD(2M) cases could also have elevations of MSA or 2OHG. These elevations did not necessarily suggest a different diagnosis.

## 4. Discussion

At the start of the period during which the study cases had newborn screening (2005), the clinical diagnosis of SCADD was primarily based on biochemical testing, with the possibility of additional confirmation through fibroblast studies or genetic sequencing. At that time, cases with two common variants were often considered to have SCADD [14]. The current, emerging clinical consensus is that only cases with two rare variants should be considered as SCADD cases, those with one rare variant should be considered SCADD carriers, and those with only common variants should be considered unaffected [6]. In the case of exome sequencing, it is necessary to add the proviso that the phase for cases with two rare variants remains undetermined and that an additional pathogenic variant could have been missed through exome sequencing. Based on the current understanding, the results of this retrospective study suggest that half of the SCADD cases diagnosed by NBS in California between mid-2005 and 2013 would not now be considered to have had true SCADD. Although even “true SCADD” may be a non-disease, metabolic centers may have chosen to use emergency protocols for these patients. This emphasizes the importance of optimizing tests to determine which infants are truly at risk for clinical disease (hypoglycemia, acidosis), while not referring individuals who are carriers of only one pathogenic variant or one or two polymorphisms, who are not at risk.

Of interest, one apparent heterozygote was labeled by CLIR as true SCADD, and one case with no identified variants in *ACADS* was labeled as true SCADD. In addition, 8 of 74, or about 10% of cases, remained indeterminate after extensive testing. These 10 cases illustrate the limitations of exome sequencing, the complexity of case follow-up, and the diagnostic challenges for the metabolic centers, even when sequencing is available. In an asymptomatic cohort, and for a condition in which symptoms may never manifest, even combined DNA and second-tier biochemical testing may fail to resolve whether an infant is truly affected. Enzyme testing is not readily available for *ACADS*; the other functional test is an in vitro probe study of labeled fat metabolism in skin cells, and even this is not 100% diagnostic [15].

It is also worth remarking that the haplotype with both common variants in *cis* is itself not uncommon. Clinicians should be aware of this possibility when interpreting genetic testing results for *ACADS* and should obtain parental genotypes.

Combining the results of the initial MS/MS testing, second-tier biochemical testing, and *ACADS* genotypes from exome sequencing allowed the tuning of the second-tier test to the point that almost half of the initial positive MS/MS tests that were clinically called SCADD at the time of screening, could now be ruled out; carrying out this testing prior to referral would have reduced the number of cases requiring follow-up, and 80% of those referred would have been determined to be affected. This discussion of performance improvement leaves out the cases that were initially referred based on the MS/MS result as SCAD positive but were resolved by the metabolic center as non-disease. It might be expected that applying the CLIR tools and the second-tier testing before referral would have eliminated most of these from the referral burden on the metabolic centers.

Some NBS programs have dealt with the complications arising from overlapping MS/MS results between SCADD and SCAD carriers by eliminating SCAD from the NBS panel [8]. Decisions about which disorders to include or exclude from newborn screening are policy decisions within the individual programs. In the United States, for example, the Advisory Committee on Heritable Disorders of Newborns and Children has included SCADD as a secondary condition in the Recommended Uniform Screening Program [16]. Many states consider this recommendation significant, with the result that 28 states include SCADD in the mandatory screening program, and an additional 8 report a positive result for SCADD derived from testing for other disorders. All of the four largest states (California, Texas, Florida, and New York) include SCADD in their mandatory panel [17]. The current study is not a recommendation to screen or not to screen for SCADD.

Rather, this study focuses on the important question of minimizing the false-positive results for programs that do decide to screen for SCADD. There is the possibility that some of the babies with two rare variants may have clinical disease, and that other conditions, such as IBDD, are identified through this analyte. Given the uncertainty regarding SCADD, a reasonable clinical course would be to follow clinically only those babies identified with two rare variants in *ACADS*. To the extent that the NBS laboratory could use second-tier testing to avoid unnecessary referrals of SCAD carriers or unaffected cases, the ability of the follow-up centers to focus on the patients who need attention could be improved.

This study was conducted in a large, ethnically diverse population. Nonetheless, the samples sequenced were only those from babies identified by NBS as having SCADD. Certainly, there were many more individuals in the population who carried a single variant in *ACADS*—particularly the common variants. If the second-tier biochemical testing were available to these, it would be likely to increase the separation between the SCADD cases and the SCAD carriers, as these individuals did not have sufficient biochemical abnormalities to trigger follow-up.

Some have suggested that DNA sequencing should become the second-tier test of choice following initial positive MS/MS results. Sequencing has challenges in both cost and turn-around time that suggest that it is not a panacea. However, this study has shown that sequencing a targeted cohort can generate data that allow substantial improvement in newborn screening through the tuning of the second-tier biochemical test. Of course, sequencing data could also be useful for the follow-up of positive NBS results, particularly if they become available in a rapid time frame.

## Figures and Tables

**Table 1:** Results of *ACADS* sequencing applied to the short-chain acyl-CoA dehydrogenase (SCAD) cohort.

Clinical Category	Genetic Category	Number
SCAD	SCAD(2M)	23
	SCAD(2M1P)	4
	SCAD(2M2P)	3
SCAD(het)	SCAD(1M1P)	24
	SCAD(1M2P)	7
Not SCAD	SCAD(2P)	10
	SCAD(1P)	1
	No SCAD variants	2
Total		74

**Table 2:** Results of the Collaborative Laboratory Integrated Reports (CLIR) SCAD tool applied to the initial MS/MS profile.

Clinical Category	Genetic Category	Number	CLIR Applied to MS/MS		
			SCAD(2M)	Needs 2TT ^1^	Not SCAD(2M)
SCAD	SCAD(2M)	23	5	18	0
	SCAD(2M1P)	4	0	4	0
	SCAD(2M2P)	3	0	3	0
SCAD(het)	SCAD(1M1P)	24	0	13	11
	SCAD(1M2P)	7	0	0	7
Not SCAD	SCAD(2P)	10	0	3	7
	SCAD(1P)	1	0	1	0
	No SCAD variants	2	0	2	0
Total		74	5	44	25
Reduction in Positives					33.80%

^1^ Second-tier test suggested to provide additional information.

**Table 3:** Results of second-tier testing with CLIR results before tuning based on genotype.

Clinical Category	Genetic Category	Number	CLIR Applied to MS/MS with 2TT ^1^		
			SCAD(2M)	Uncertain	Not SCAD(2M)
SCAD	SCAD(2M)	23	21	2	0
	SCAD(2M1P)	4	4	0	0
	SCAD(2M2P)	3	3	0	0
SCAD(het)	SCAD(1M1P)	24	10	2	12
	SCAD(1M2P)	7	3	1	3
Not SCAD	SCAD(2P)	10	0	0	10
	SCAD(1P)	1	1	0	0
	No SCAD variants	2	1	0	1
Total		74	43	5	26
PPV		40.50%	62.50%		
Reduction in Positives					35.1%

^1^ Second-tier test. PPV: positive predictive value.

**Table 4:** Results of second-tier testing with CLIR results after tuning based on genotype.

Clinical Category	Genetic Category	Number	CLIR Applied to MS/MS with 2TT ^1^		
			SCAD(2M)	Uncertain	Not SCAD(2M)
SCAD	SCAD(2M)	23	21	2	0
	SCAD(2M1P)	4	4	0	0
	SCAD(2M2P)	3	3	0	0
SCAD(het)	SCAD(1M1P)	24	1	6	17
	SCAD(1M2P)	7	0	0	7
Not SCAD	SCAD(2P)	10	0	0	10
	SCAD(1P)	1	0	0	1
	No SCAD variants	2	1	0	1
Total		74	30	8	36
PPV		40.50%	78.90%		
Reduction in Positives					48.6%

^1^ Second-tier test.

## Supplementary Materials

Supplementary materials can be found at https://www.mdpi.com/2409-515X/6/2/41/s1.
