# Supplementary material for: Genomic Analysis of Historical Cases with Positive Newborn Screens for Short-Chain Acyl-CoA Dehydrogenase Deficiency Shows That a Validated Second-Tier Biochemical Test Can Replace Future Sequencing
Source: Int J Neonatal Screen. 2020 May 26;6(2):41. doi: 10.3390/ijns6020041 (PMC7423011; doi:10.3390/ijns6020041)
Supplement: Supplementary file 1 [file IJNS-06-00041-s001.pdf]

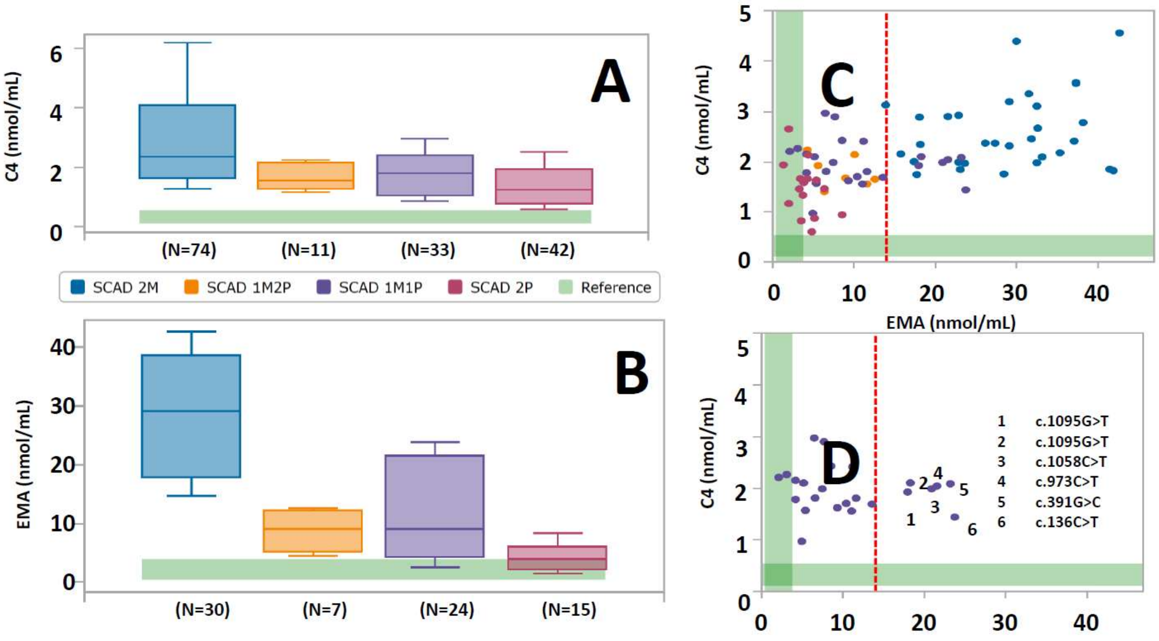

Figure S1. Comparison of C4 and EMA in SCAD(2M), SCAD(1M2P), SCAD(1M1P), and SCAD(2P). Panels A and B are reference distributions for C4 and EMA from CLIR. Panel C is the scatter plot of the data from the study. Panel D is the same plot for the SCAD(1M1P) subset. The six marked are compound heterozygotes between c.625G>A and the variants listed.

Table S1. *ACADS* variants found in study population.

ID	Genotype	# of ACADS:c.625G>A Copies	# of ACADS:c.511C>T Copies	ACADS Variants Flagged by AA10.1 Pipeline (Counts)
SCAD_01	No SCAD Variants	0	0	
SCAD_02	SCAD(1M1P)	1	0	c.889C>T (1),
SCAD_03	SCAD(1M1P)	1	0	c.326_328del (1),
SCAD_04	SCAD(1M1P)	1	0	c.934-2A>G (1),
SCAD_05	SCAD(1M1P)	1	0	c.409C>T (1),
SCAD_06	SCAD(1M1P)	1	0	c.1058C>T (1),
SCAD_07	SCAD(1M1P)	1	0	c.1138C>T (1),
SCAD_08	SCAD(1M1P)	1	0	c.1138C>T (1),
SCAD_09	SCAD(1M1P)	1	0	c.1095G>T (1),
SCAD_10	SCAD(1M1P)	1	0	c.529T>C (1),
SCAD_11	SCAD(1M1P)	1	0	c.529T>C (1),
SCAD_12	SCAD(1M1P)	1	0	c.47-6C>A (1),
SCAD_13	SCAD(1M1P)	1	0	c.1130C>T (1),
SCAD_14	SCAD(1M1P)	1	0	c.136C>T (1),
SCAD_15	SCAD(1M1P)	1	0	c.1095G>T (1),
SCAD_16	SCAD(1M1P)	1	0	c.1192C>T (1),
SCAD_17	SCAD(1M1P)	1	0	c.461T>G (1),
SCAD_18	SCAD(1M1P)	1	0	c.508G>A (1),
SCAD_19	SCAD(1M1P)	1	0	c.973C>T (1),
SCAD_20	SCAD(1M1P)	1	0	c.268G>A (1),
SCAD_21	SCAD(1M1P)	1	0	c.322G>A (1),
SCAD_22	SCAD(1M1P)	1	0	c.529T>C (1),
SCAD_23	SCAD(1M1P)	1	0	c.391G>C (1),
SCAD_24	SCAD(1M1P)	1	0	c.529T>C (1),
SCAD_25	SCAD(1M1P)	1	0	c.529T>C (1),
SCAD_26	SCAD(1M2P)	2	0	c.973C>T (1),
SCAD_27	SCAD(1M2P)	2	0	c.934-11G>A (1),
SCAD_28	SCAD(1M2P)	2	0	c.1147C>T (1),
SCAD_29	SCAD(1M2P)	2	0	c.973C>T (1),
SCAD_30	SCAD(1M2P)	2	0	c.250G>A (1),
SCAD_31	SCAD(1M2P)	2	0	c.1147C>T (1),
SCAD_32	SCAD(1M2P)	2	0	c.280G>C (1),
SCAD_33	SCAD(1M2P)	2	0	c.136C>T (1),
SCAD_34	SCAD(1P)	1	0	
SCAD_35	SCAD(2M)	0	0	c.529T>C (2),
SCAD_36	SCAD(2M)	0	0	c.529T>C (2),
SCAD_37	SCAD(2M)	0	0	c.529T>C (2),
SCAD_38	SCAD(2M)	0	0	c.529T>C (2),
SCAD_39	SCAD(2M)	0	0	c.319C>T (2),
SCAD_40	SCAD(2M)	0	0	c.529T>C (1), c.1039A>G (1),
SCAD_41	SCAD(2M)	0	0	c.1031+1A>G (1), c.1130C>T (1),
SCAD_42	SCAD(2M)	0	0	c.981_983del (1), c.1192C>T (1),
SCAD_43	SCAD(2M)	0	0	c.529T>C (1), c.988C>T (1),
SCAD_44	SCAD(2M)	0	0	c.1195C>T (2),
SCAD_45	SCAD(2M)	0	0	c.529T>C (2),
SCAD_46	SCAD(2M)	0	0	c.529T>C (2),
SCAD_47	SCAD(2M)	0	0	c.529T>C (2),
SCAD_48	SCAD(2M)	0	0	c.1195C>T (2),
SCAD_49	SCAD(2M)	0	0	c.529T>C (1), c.988C>T (1),
SCAD_50	SCAD(2M)	0	0	c.508G>A (1), c.673G>C (1),
SCAD_51	SCAD(2M)	0	0	c.529T>C (2),
SCAD_52	SCAD(2M)	0	0	c.529T>C (2),
SCAD_53	SCAD(2M)	0	0	c.529T>C (1), c.988C>T (1),
SCAD_54	SCAD(2M)	0	0	c.1195C>T (2),

SCAD_55	SCAD(2M)	0	0	c.682G>A (1), c.1147C>T (1),
SCAD_56	SCAD(2M)	0	0	c.529T>C (1), c.941_949del (1),
SCAD_57	SCAD(2M)	0	0	c.529T>C (2),
SCAD_58	SCAD(2M1P)	1	0	c.203C>T (1), c.391G>C (1),
SCAD_59	SCAD(2M1P)	1	0	c.268G>A (1), c.1192C>T (1),
SCAD_60	SCAD(2M1P)	1	0	c.529T>C (1), c.1067C>T (1),
SCAD_61	SCAD(2M1P)	1	0	c.136C>T (1), c.934-5T>A (1),
SCAD_62	SCAD(2M2P)	2	0	c.596C>T (2),
SCAD_63	SCAD(2M2P)	2	0	c.868G>C (2),
SCAD_64	SCAD(2M2P)	2	0	c.136C>T (1), c.1153G>T (1),
SCAD_65	SCAD(2P)	2	0	
SCAD_66	SCAD(2P)	2	0	
SCAD_67	SCAD(2P)	2	0	
SCAD_68	SCAD(2P)	2	0	
SCAD_69	SCAD(2P)	2	0	
SCAD_70	SCAD(2P)	2	0	
SCAD_71	SCAD(2P)	2	0	
SCAD_72	SCAD(2P)	2	0	
SCAD_73	SCAD(2P)	2	0	
SCAD_74	SCAD(2P)	2	0	
